# Frontier molecular orbital weighted model based networks for revealing organic delayed fluorescence efficiency

**DOI:** 10.1038/s41377-024-01713-w

**Published:** 2025-02-10

**Authors:** Zhaoming He, Hai Bi, Baoyan Liang, Zhiqiang Li, Heming Zhang, Yue Wang

**Affiliations:** 1 Jihua Laboratory, Foshan, Guangdong Province PR China; 2 State Key Laboratory of Supramolecular Structure and Materials, College of Chemistry, Jilin University, Changchun, PR China; 3 Jihua Hengye Electronic Materials CO. LTD, Foshan, Guangdong Province PR China

**Keywords:** Optical materials and structures, Physics

## Abstract

Free of noble-metal and high in unit internal quantum efficiency of electroluminescence, organic molecules with thermally activated delayed fluorescence (TADF) features pose the potential to substitute metal-based phosphorescence materials and serve as the new-generation emitters for the mass production of organic light emitting diodes (OLEDs) display. Predicting the function of TADF emitters beyond classic chemical synthesis and material characterization experiments remains a great challenge. The advances in deep learning (DL) based artificial intelligence (AI) offer an exciting opportunity for screening high-performance TADF materials through efficiency evaluation. However, data-driven material screening approaches with the capacity to access the excited state properties of TADF emitters remain extremely difficult and largely unaddressed. Inspired by the fundamental principle that the excited state properties of TADF molecules are strongly dependent on their D-A geometric and electronic structures, we developed the Electronic Structure-Infused Network (ESIN) for TADF emitter screening. Designed with capacities of accurate prediction of the photoluminescence quantum yields (PLQYs) of TADF molecules based on elemental molecular geometry and orbital information and integrated with frontier molecular orbitals (FMOs) weight-based representation and modeling features, ESIN is a promising interpretable tool for emission efficiency evaluation and molecular design of TADF emitters.

## Abstract (web summary)

An electronic structure-infused deep-learning neural network based on frontier molecular orbitals representation is constructed to predict the emission efficiencies of thermally activated delayed fluorescence (TADF) emitters.

## Graphical abstract

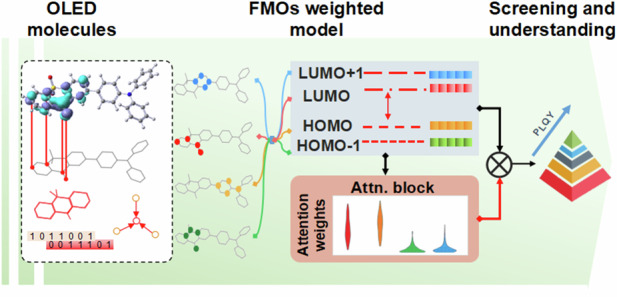

## Introduction

Organic light-emitting diodes (OLEDs) have garnered significant attention due to their potential in various display and lighting applications. A crucial factor in their efficiency and longevity is the choice of emitter. Purely organic molecules with thermally activated delayed fluorescence (TADF) features perform reverse intersystem crossing (RISC) process, converting triplet excited state energy into light emission^1–3^. Leveraging the RISC process, OLED devices with TADF emitters can achieve 100% internal quantum efficiency (IQE). Over the past decade, cost-effective organic TADF materials have emerged as strong contenders to replace metal-based phosphorescent materials that currently dominate the active matrix OLED (AMOLED) display manufacturing industry, offering the dual benefits of being free from noble metals and achieving high internal quantum efficiency of electroluminescence^4,5^. Despite significant investment from both industrial and academic sectors, the experimental trial-and-error method remains lengthy, with extremely limited success in high-performance TADF emitters suitable for industrial applications.

The design principle of high-performance TADF emitters is drawn from the underlying luminescent process theories. To promote and accelerate the RISC process, a critical factor is maintaining a sufficiently small singlet–triplet splitting energy (Δ*E*_ST_) between the lowest singlet (S_1_) and triplet (T_1_) excited states, which can be achieved by minimizing the overlap between the highest occupied molecular orbital (HOMO) and the lowest unoccupied molecular orbital (LUMO) of organic molecules. Typically, TADF molecules possess either intra or inter-molecular charge transfer (ICT) characteristics and comprise electron donor (D) and electron acceptor (A) moieties. For ICT molecules, their HOMO and LUMO are localized in the D and A groups, respectively. The fundamental molecular design strategy for TADF emitters, thus, centers on creating a spatial separation architecture for the D and A groups and ensuring minimal shared chemical bonds and atoms between the two groups. Following this vague principle, a huge number of TADF molecules have been synthesized but only a small proportion of materials displayed high electroluminescent (EL) efficiency with ideal stability.

Generally, obtaining a desired TADF molecule often requires laborious trial-and-error. A non-chemical lab experimental approach that accurately predicts the EL efficiency of TADF molecules could accelerate the optimization process. The considerable reductions in organic synthesis, purification, photophysical characterization, device fabrication, and performance assessment of the candidate molecules align with the goal of “green chemistry”. Determining the dominating structural features of TADF molecules during their complex transition between ground state and excited state remains a challenge. Although density functional theory (DFT) calculations can offer predictions on the electronic structure, energy level, and photophysical information of organic molecules, such as Δ*E*_ST_, photoluminescence quantum yield (PLQY), the lifetime of the excited state, and emission maximum, the computation is often time-consuming and may deviate from the actual values, especially for complex D-A structured molecules. With recent advances in deep learning (DL) techniques, there is a considerable potential for a more reliable approach to predict the excited state properties of TADF molecules.

Data-driven DL approaches have been proven effective in chemistry and chemical material studies, especially the investigation of structure−property relationships^6–10^. An ideal DL-based organic material design approach should have: (i) an accurate molecular representation for the input and (ii) an interpretable model that rationally associates the critical structure elements with molecular properties. Although simplified molecular input line entry systems (SMILES) and molecular fingerprints are commonly used for molecular representations^11–13^, they often overlook key elements influencing certain properties. The application of existing DL models such as convolutional neural networks (CNNs)^14,15^, recurrent neural network (RNN)^16^, and graph neural networks (GNN)^17,18^ in the fields of chemistry or chemical material without modifications is usually inappropriate as these models were established for other domains. Another significant drawback of purely data-driven DL approaches in organic material design is their lack of interpretability, leading to an inability to derive property insights associated with material performances.

The focus in pursuing an efficient DL-guided performance prediction and design method for TADF materials lies in molecular feature representation and DL model optimization^19^. Luminescent properties of TADF molecules arise from both their molecular geometry and electronic structures, but electronic structures have been long overlooked in previous AI prediction attempts of TADF molecule properties. The excited property of a TADF molecule is predominantly influenced by its D and A groups, particularly certain key atoms and chemical bonds. Thus, molecular representation and model optimization in the current study are based on the critical molecular geometrical and electronic features of the D and A groups of TADF molecules.

In this contribution, we developed an “Electronic Structure-Infused Network (**ESIN**)” for data-driven emission efficiency prediction and TADF emitter design. Built on the basis of elemental chemical features and a limited amount of data including reported experimental results and theoretical calculations of recently published TADF molecules, **ESIN** is endowed with the ability to predict the PLQYs of TADF molecules through an essential “frontier molecular orbitals (**FMOs**) weight-based representation and modeling” feature. Two to five atoms exhibiting the largest weight in HOMO-1, HOMO, LUMO, and LUMO + 1 are selected as the topological centers. Subsequently, multiple substructures based on each topological center were sampled via a D and A groups-based method named Sampling and Aggregate (**SAGE**) to generate local representations of molecular orbitals. The model also integrates global information from the molecular fingerprints associated with these substructures to construct a comprehensive representation of the molecular orbitals. It is worth noting that each generated representation only correlates with the D or A group. More importantly, considering the crucial role of coupling between the vibrational energy level and electronic energy level, the maximum harmonic frequencies and corresponding vibration vectors are also integrated into the molecular representation. To strengthen model interpretability, attention mechanism generates a ratio representing the contribution of each FMO on the predicted PLQY of the input TADF molecule^20,21^. The significance of **ESIN** in predicting PLQY and recommending molecular design of TADF emitters has been experimentally verified.

## Results

### Designs of frontier molecular orbital weighted model

**Fig. 1:**
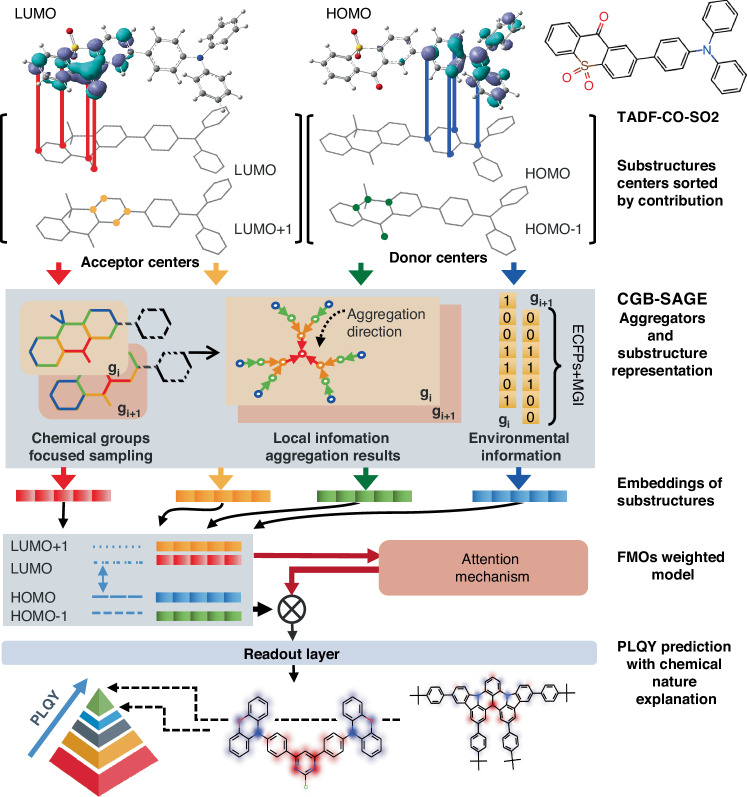
Construction process of ESIN. Illustration of the whole network that contains the generation of embeddings of the corresponding substructures of the FMOs, and the chemical nature explanation of the prediction

Our strategy for molecular representations prioritizes the key atoms in D and A groups, which are selected, screened, and inferred based on the elemental chemical features and low-level theoretical calculations of the D-A molecules. Figure 1 outlines the **ESIN** conception and PLQY prediction process. TADF-CO-SO2 (2-(4-(diphenylamino)phenyl)-9*H*-thioxanthen-9-one-10,10-dioxide) molecule was used as an example to illustrate the principle of generating molecular substructure representations, embeddings, and attention mechanism. Semi-empirical level (PM7) calculations provide molecular geometry and electronic structures^22,23^. For TADF-CO-SO2, four substructures were determined based on the contributions of atoms to FMOs including HOMO-1, HOMO, LUMO and LUMO + 1, respectively.

**Fig. 2:**
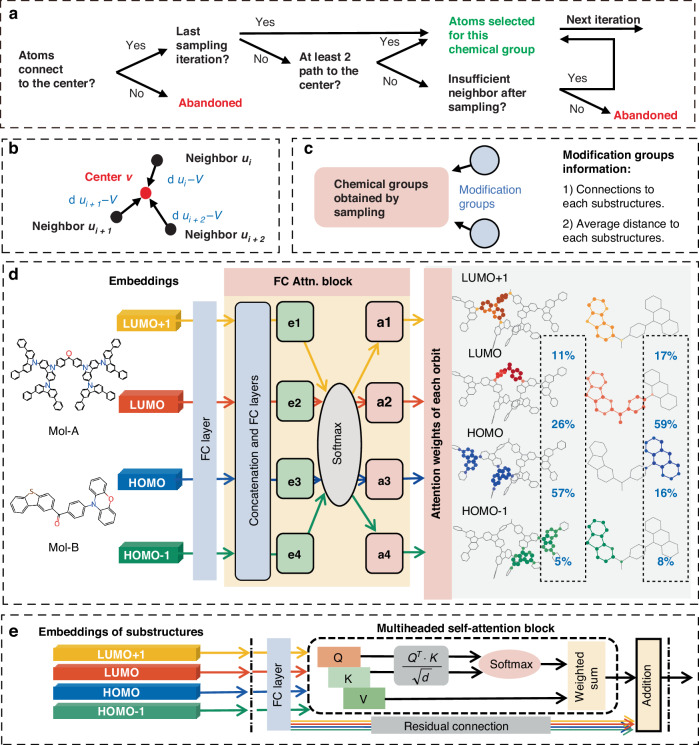
Advanced representation methods and attention mechanisms in ESIN for analyzing substructure contributions. **a** Chemical group-based sampling rules. **b** Distance information incorporated into the aggregators. **c** Components included in the modification groups information. **d** FC attention block and attention weights distribution of corresponding electronic structures of two TADF molecules. The colors of atoms present the contribution rates of atoms to the FMOs. **e** Attention mechanism with transformer block

A few atoms contributing significantly to FMOs were defined as the cornerstones for each substructure. In a substructure, the atom with the most substantial contributions to the FMO local center atom (LC-Atom) was adopted as the center (red node) of an aggregator. Such aggregation information on all the local sampling centers is then combined with the extended-connectivity fingerprints (ECFPs) of the corresponding substructure to build the final embeddings of each FMO^13^. The LC-Atom-centered aggregator was utilized as a topological framework to construct molecular representations infused with the critical information relevant to FMOs. To ensure the molecule representation with adequate chemical connotation, a Chemical Groups-Based Sampling and Aggregate (**CGB-SAGE**) method was introduced to generate FMO embeddings. The SAGE method, originally proposed for the graph neural networks, produces node embeddings with a nonselective feature extraction of neighbors in specified “search depths”^24^. As the CGB-SAGE process illustrated in Fig. 1, for an acceptor group g_i_, starting from the LC-Atom, repeated neighbor sampling yields progressively larger regions (red → orange → green → blue). After obtaining the topological framework through sampling, each iteration of the aggregator collects the information from adjacent neighbors towards the center, in the order of (blue → green → orange→ red). Through this iterative process, the information from the acceptor area is aggregated to the LC-Atom. Notably, the CGB-SAGE approach developed in this study is tailored based on aromatic rings within D or A group, and in alignment with the FMO distribution features. Four criteria in each iteration of the CGB-SAGE are detailed in Fig. 2a, where neighbors are identified by assessing if the atoms have at least two distinct pathways to the sampling center. The semi-empirical level calculation results reveal that hydrogen atoms have negligible contribution to HOMO-1, HOMO, LUMO, and LUMO + 1. Hence, the hydrogen atoms are excluded during the sampling process.

1 $${h}_{v}^{(k)}={\sigma }_{out}\left(\frac{1}{n}\left[\mathop{\sum }\limits_{i=1}^{n-1}\sigma ({h}_{{u}_{i}}^{(k-1)},{d}_{{u}_{i}-v})+\sigma ({h}_{v}^{(k-1)},{d}_{v-v})\right]\right)$$

Moreover, both FMO information and 3D coordinate relationship are incorporated into the aggregator of the CGB-SAGE. As depicted in Eq. 1, σ_*out*_ and σ are two fully connected (FC) layers, in the iteration process the center and the neighbors share the FC layer. *h*_*v*_ represents the features of center atom *v* and *h*_*ui*_ symbolizes the features of the neighbors of atom *v*. *h*_*v*_ and *h*_*ui*_ are treated separately in each iteration. *d*_*v*-*v*_ represents the distance for the center atoms themselves and is defined as 0, while the *d*_*ui-v*_ is the distance from the neighbor atom *u*_*i*_ to the center atom *v* (Fig. 2b). The initial values for the features *h*_*v*_ and *h*_*ui*_ are defined based on the basic information of each atom’s essential chemical properties including FMOs, harmonic frequencies and vibration vectors (Table 1). The coupling between electron and vibrational transitions significantly impacts the properties of excited state molecules. The maximum harmonic frequencies that are associated with ground state and the corresponding vibration vectors are adopted as the molecular vibration information in the atom representation^25,26^. Experimental results showed that our model, infused with electronic structure and vibration information, excels in establishing the correlations and causalities between structures and properties of TADF molecules (Table S1 in Supporting Information).

**Table 1:** Atom representation of the ESIN

Feature	Description	Size
Atom type	Embeddings of the type of atom (e.g., C, N, O, S).	32
Harmonic frequencies	5 vibration frequencies (cm^−1^) with the largest intensity.	5
Vibration vector	Vibration vector of corresponding frequencies.	15
Relative position to the sampling center	Distance to the sampling centers in Cartesian coordinates.	3
Chemical bonds	Number of bonded atoms.	1
Cyano	Whether the cyano group is connected.	1

To provide a more comprehensive description of the substructure’s structural information, the model concatenates the embeddings of the local sampling centers with ECFPs. The ECFPs used are fixed-length binary encodings that describe the local environment around each atom in the substructure with in a certain radius (see the Materials and Methods section). In addition, the Modification Groups Information (MGI) is encoded and integrated with the environmental information (Fig. 2c), including the number and the average distances from the attached key groups to the LC-Atom within the substructure. Although the ECFPs are typically effective for binary numerical encoding of small molecules in other studies^27–29^, the ECFPs proposed in the current work are capable of describing fragments of large molecules due to the fact that the rational environmental information of the sampling centers was adopted.

Two model structures are used and compared to merge the embeddings of the four FMOs (HOMO-1, HOMO, LUMO, and LUMO + 1). One architecture is referred to as the FC Attention Block, while the other is a generic Transformer Encoder, namely Transformer Output Layer. The transformer output layer comprises a layer of multiheaded self-attention (MSA) block and a residual connection (Fig. 2e)^20^.

The Mol-A typically exhibits a significantly larger molecular size and atom count compared with Mol-B (Fig. 2d). Despite this, during the sampling process, relatively similar-sized substructures have been generated and used for both molecules. The embeddings of substructures are concatenated and fed to the FC attention block to generate attention weights **(**Fig. 2d). The FC Attention Block consists of two FC layers, which generate factors e1-e4 to quantify the influence of each molecular orbital on the prediction. These factors are normalized using the softmax function to obtain attention weights a1-a4, which are then used to compute a weighted sum of the representations of the four molecular orbitals to obtain the overall molecular orbital representation. These weights, representing both electronic structures and spatial structures of the TADF molecules, are critical for the understanding of the PLQY prediction mechanism. Orbitals with larger attention weights have a more pronounced effect on prediction outcomes, indicating their significant contribution to a TADF molecule’s PLQY value. The PLQY prediction is exported by the READOUT function with a sum pooling layer and two FC layers. The prediction results underscore that the **ESIN** approach has been successfully focused on the key structural elements that govern the PLQYs of TADF molecules, omitting subsidiary structural elements. This is especially vital for the studies of TADF molecules with large sizes, complex structures, and multiple functional groups.

For the Transformer Output Layer, the MSA block generates the attention weights for inter-molecular orbitals (Fig. 2e). The model generates query (Q), key (K), and value (V) vectors for each of the four orbitals using a FC layer. These Q and K vectors are then used to compute association factors between molecular orbitals, which are normalized by the softmax function to produce the attention weights. Differing from the attention weights in the FC Attention Block, which focus on the influence of each molecular orbital on the prediction, the attention weights generated in the Transformer Encoder emphasize the inter-FMOs associative and influential characteristics. Each FMO is assigned four attention weights, which are used in the weighted sum of V vectors to generate the adjusted representation for each FMO. Finally, the representations of 4 FMOs are combined to form the overall molecular orbital representation, which is then employed to predict the PLQY through the READOUT function.

### TADF experimental dataset and performance of models

**Fig. 3:**
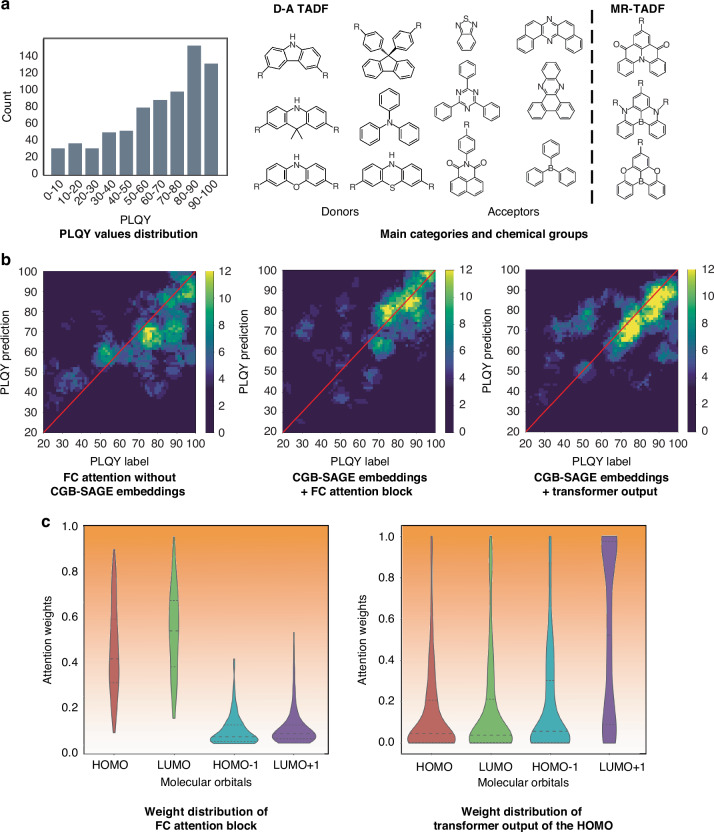
Experimental results based on ESIN. **a** Details of TADF molecules in our data set. **b** A representation visualization comparison for different **ESIN** variants. The color of each pixel is plotted according to the number of data points existing within the radius of 6. **c** Comparison of the statistical distributions of attention weights corresponding to the 4 molecular orbitals. The dotted and dashed lines represent the quartiles

The PLQYs of doped thin films (858), crystal (1) and solutions (80) dataset are drawn from recently reported photophysical results of TADF molecules. Out of 1085 initial data entries with PLQY values for doped films and/or solutions, 939 TADF molecules were deemed valid in format. All TADF emitters in the data set are D-A-type molecules, encompassing well-known donor and acceptor groups. It is worth noting that some multiple resonance TADF (MR-TADF) molecules are also included^30–34^. These molecules were randomly divided into a training set and a test set in an 8:2 ratio. Figure 3a provides an overview of the data set used in this study. The preponderance of reported TADF molecules showcased exemplary electroluminescent performance, accounting for the dataset’s tilt towards relatively high PLQY levels.

The prediction results, derived from three **ESIN** variants are compared in Fig. 3b. The color distribution reflects the performances of the models. Evaluation results highlighted the efficacy of the CGB-SAGE blocks in enhancing the performance of **ESIN** over representations generated exclusively by ECFPs. Comparing the **ESIN** using the FC attention block to that using the transformer output layer, the former resulted in a more balanced prediction performance across the spectrum of reported PLQY values, while the latter excels in PLQY values above 60. These findings are in accordance with the lower mean absolute percentage error of the **ESIN** with FC attention, as outlined in Table S3 in Supporting Information. The diminished prediction accuracy of **ESIN** with a transformer output layer for lower PLQY values can be associated with the imbalance of PLQY distribution in the training data, especially considering the model’s extensive parameter count (over 2 million). In contrast, the **ESIN** with a FC attention block only contains about 350 K parameters, suggesting its higher suitability for a relatively smaller training set. Comprehensive cross-model comparisons of the PLQY prediction performances also reveal that **ESIN** with FC attention block is on par with the reported leading models (Fig. S1)^35–39^. We evaluated our model using the results of the bisection method. A threshold of 85% is adopted, which can cover most high-quality molecules, and the model achieved an accuracy of 70% and a recall rate of 40% in predicting experimental PLQY values that are greater than the threshold in test set. The results demonstrate that the model in this study is efficient for quickly screening out high-PLQY TADF molecules.

### Chemical intuition and molecular design insights of the model

Four FMOs are involved in **ESIN** to explore the influence of electronic structures on the PLQY values of TADF molecules. The results originated from the FC attention block revealed that the embeddings of HOMO and LUMO dominate the attention values. The attention weights obtained from the transformer output layer show that the attention weight values associated with LUMO + 1 and HOMO exceed those associated with the other FMOs (Fig. 3c). These findings demonstrate the important role of HOMO, LUMO and LUMO + 1 in the TADF emission process. The distribution of attention weights in **ESIN** with transformer output layer is consistent with the understanding of the RISC process in partial TADF molecules, which is associated with the higher triplet excited energy levels. Systematic statistical analysis on the distribution of attention weights of the four FMOs reveals that for a TADF molecule, one or two of the three FMOs, including HOMO, LUMO, and LUMO + 1, exhibit larger attention weight(s).

Meanwhile, HOMO-1 consistently registers the smallest attention weight. For a series of TADF molecules sharing the same D-A framework but featuring varied modified groups, each molecule exhibits an identical or similar distribution pattern for the attention weight parameters of the four FMOs. These results perfectly align with the elementary principles of TADF molecules: (i) the excited state results in the electronic transition from HOMO to LUMO or LUMO + 1; (ii) the molecules with analogous D-A structures possess similar emission properties.

Three series of TADF molecules with designated D-A frameworks are selected from the database as examples and illustrated in Fig. S2. For the TADF molecules with rigid oxygen bridged boron containing acceptor, those with elevated PLQYs also exhibit relatively lower variances in the attention weights of four FMOs. A similar trend – a rise in PLQYs concurrent with a reduction in attention weight variance – was also observed in the results of 2,4,6-triphenyl-1,3,5-triazine (TRZ) acceptor-based TADF molecules with a D-A-D arrangement feature. Figure S2c illustrates two sets of molecules with different 1,8-naphthalimide (NAI) based acceptors. Despite a discrepancy in slope of the trend line between the two groups, both exhibit a remarkable correlation between PLQY and variance of attention weights. The information on TADF molecules with NAI-based acceptors in the distribution map is detailed in Table S4.

**Fig. 4:**
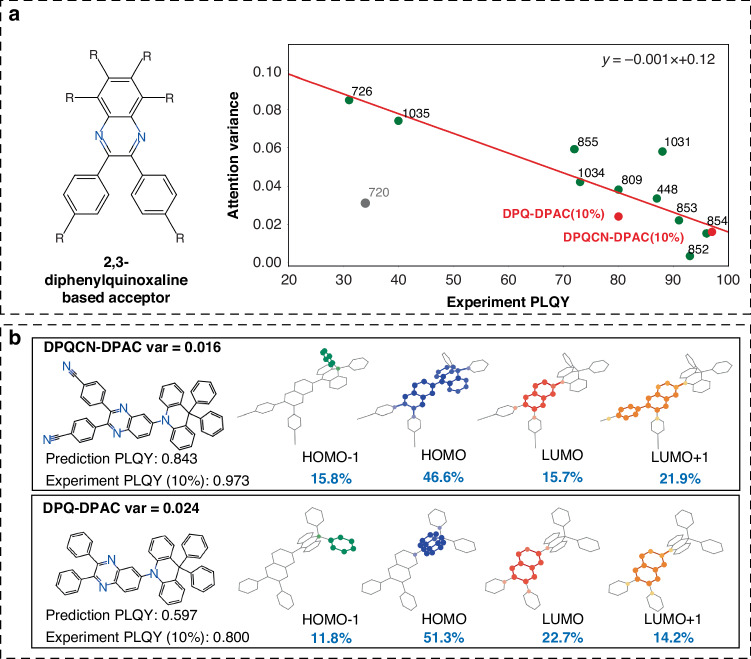
Representative samples. **a** Scatter diagram of the relationship between the variance of attention weights of corresponding orbitals and the experiment PLQYs. The linear fit trend line in the figure was calculated using the Ordinary Least Squares (OLS) method with the effective samples. **b** The experiment result of the synthesized TADF molecules DPQ-DPAC and DPQCN-DPAC

The distribution of attention weight values aligns closely with the PLQYs, especially for a series of TADF molecules bearing a similar D-A framework (Fig. 4). Figure 4a presents the diphenylquinoxaline-based molecules collected in the data set (for details, please see Table S5). Since most of the PLQYs in the dataset were obtained from doped films and solutions, the trend line was drawn excluding the point from the crystalline sample, which has lower PLQY and was marked in gray in Fig. 4a. The PLQY values are inversely correlated with the variance of attention weights. Although the relationship is not sufficiently robust due to the limited data, this trend may allow us to quickly filter emitters and suggest molecular modification strategy.

To validate the interpretability of **ESIN**, two new diphenylquinoxaline-based molecules DPQ-DPAC and DPQCN-DPAC (Fig. 4b and Table S6) were synthesized and experimentally accessed (details in Supporting Information). The PLQY values of DPQ-DPAC and DPQCN-DPAC were accurately predicted, and the higher experimental value of PLQY of the former corresponded with its lower variance of model-generated attention weights for the four FMOs, as determined by the model. The structure and the attention weights of the synthesized molecules are depicted in Fig. 4b. A shift in attention weights is evident upon modifying the acceptor. The cyano group in DPQCN-DPAC increases the attention weight of the LUMO + 1 and suppresses the variance of attention weights, which corresponds with the enhancement in PLQY value. Based on the model verification, the ability of **ESIN** to evaluate the luminescent potential of TADF molecules can be attributed to the capacity to investigate the molecular structure-dependent FMO characteristics and potential, which are associated with PLQYs of TADF molecules. Consequently, **ESIN** emerges as an innovative tool for proposing high PLQY TADF molecules, extending beyond traditional molecular design strategies.

For designing a new efficient molecule, we typically start with a reported core framework and explore different combinations and modifications to optimize the electronic distribution. The trained ESIN model, which can filter out molecules with lower PLQY, provides an effective method for optimizing design. For a target D-A structure, the first step involves analyzing the relationship between attention weights and PLQY to fit distribution patterns. Next, by identifying the substructures that have the greatest impact on the distribution patterns, we can focus on the key substructures. Finally, through model predictions and experimental feedback, we refine the distribution pattern model to find the best modification sites, thus rapidly identifying efficient TADF materials.

## Discussion

To pave the way for an AI-based PLQY prediction and molecular design approach for TADF emitters, **ESIN** was established. Originating from the fundamental understanding that the excited state properties of TADF molecules are intimately associated with their D-A geometric and electronic structures, we endeavored to imbue **ESIN** with a “chemistry mind” capable of comprehensively evaluating the structure-property relationship of TADF molecules. The model, grounded in the principles of FMO theory, significantly bolsters the explainability of the DL framework. The molecular representations in this study prioritize the crucial atoms in the D or A groups that dominate the excited state properties of TADF molecules. A few of the atoms that predominately influence HOMO-1, HOMO, LUMO, and LUMO + 1 are adopted as key elements, and the coupling between vibrational and electronic transitions was woven into the molecular representations. This is particularly advantageous for characterizing molecules with multiple donor and acceptor groups, differing from other models that rely on expanding the sampling range when representing each atom to enhance comprehension of the entire molecule which often leads to an exponential surge in computational requirements.

This approach introduces a new paradigm in efficiently characterizing organic luminescent molecules. The integration of attention mechanisms allows for a nuanced understanding of the relative importance of different segments in the luminescence pathway. By examining the attention distribution shifts across structurally varied molecules of the same system and correlating these with experimental PLQY results, we can discern patterns that guide us towards designing candidates with heightened PLQY. In the case of the diphenylquinoxaline-based molecules, by modifying the group of the candidates with the highest contribution to the LUMO + 1 orbital, we engineered a subtle yet profound shift in the model’s sensitivity from primarily focusing on the HOMO to emphasizing the LUMO + 1. This deliberate manipulation, informed by the model’s insights, led to the synthesis of TADF molecules exhibiting enhanced efficiency.

Currently, while large-scale models are rapidly evolving, there remains a scarcity of datasets related to TADF materials that are sufficiently extensive to warrant the training of a TADF-specific large-scale model. Even when trained with a relatively limited dataset, **ESIN** demonstrates superior interpretability and PLQY prediction capacity, offering invaluable insights for high-performance TADF emitter design. With the accumulation of experimental data, our model stands as an interpretable and efficient molecular representation approach, poised for application in future large-scale deep learning studies aimed at elucidating other photoelectronic properties of organic materials.

So far, available TADF training datasets are still limited, and the standardization across different datasets is hard to be achieved. Therefore, inaccuracies in the training data should be expected, which might affect the predictions. However, it is anticipated that upon new available data increasing, we can gradually refine and improve our molecular orbital model that will meet the demand for accurate predictions of PLQYs.

It is worth to note that at present, the model reported in this study may not be applied to other high-performance electroluminescent materials such as iridium or platinum based phosphorescent complexes. Therefore, the model should be modified or rebuilt in the future.

## Materials and methods

### Training platform and setting

The ESIN is developed in TensorFlow^40^, with training carried out via mini-batch stochastical gradient descent using the Adam optimizer^41^. The batch size is set to 32 and the learning rate is designated as 0.01 during training. The loss value of the model is obtained by summing the L2 loss and the regularization term losses^42^. Comprehensive hyperparameters are detailed in the Supporting Information (Table S2). Weight initialization for convolution operations and output layers is executed using the built-in initializer of the Tensorflow.

A NVIDIA GeForce RTX 3090 Ti GPU was used for model training and prediction. After the necessary semi-empirical calculations results were prepared, the model can be fully trained within 24 h based on our dataset. For each new molecule, the prediction time is very fast, typically taking less than one second.

### ECFPs

In the **ESIN** running process, the ECFPs are calculated using RDKit^43^ tools with feature-based invariants. The length of the ECFPs for each substructure is 2048. For computing the integer encoding of ECFPs, we set the sampling radius to 2. The radius determines the depth of the connectivity information captured, which refers to the number of bond hops away from the central atom. A larger radius includes more distant neighbors and more complex structural information. Due to the limited number of molecules in our dataset, we haven’t selected larger radius. To prevent non-convergence, in **ESIN** the employed lengths of ECFPs for FC attention block and transformer output layer patterns are 2048 bits and 300 bits, respectively.

### Materials purification

All materials used in this study were purified by sequentially crystallization, column chromatography and two times vacuum sublimation.

### Photoluminescence measurements

The steady PL spectra and PLQYs were obtained with an Edinburgh FLSP920 fluorescence spectrophotometer equipped with a xenon arc lamp (Xe750) and integrating sphere. PLQY values were measured by Hamamatsu absolute PL Quantum yield spectrometer C11347 with excitation wavelength of 365 nm. The samples were measured after N_2_ bubbling for 5 min.

### Theoretical calculations

The stablest geometric conformers for the molecules in the database were optimized by CREST driven xTB progam package 6.4.1version at GFN1 level^44–47^. Based on the stablest conformers the vibrational normal modes were achieved using PM7 as a semiempirical density functional thorery method supported by Gaussian 09 D01 version^48^. The orbital compositions were calculated by Multiwfn program using Hirshfeld method based on the converged electronic wavefunctions supported by Gaussian 09^49–53^.

## Supplementary information

Supplementary Information for Frontier Molecular Orbital Weighted Model Based Networks for Revealing Organic Delayed Fluorescence Efficiency

## References

[1] Uoyama, H. et al. Highly efficient organic light-emitting diodes from delayed fluorescence. *Nature* **492**, 234–238 (2012). 23235877 10.1038/nature11687

[2] Hirata, S. et al. Highly efficient blue electroluminescence based on thermally activated delayed fluorescence. *Nat. Mater.* **14**, 330–336 (2015). 25485987 10.1038/nmat4154

[3] Kondo, Y. et al. Narrowband deep-blue organic light-emitting diode featuring an organoboron-based emitter. *Nat. Photonics* **13**, 678–682 (2019).

[4] Baldo, M. A. et al. Highly efficient phosphorescent emission from organic electroluminescent devices. *Nature* **395**, 151–154 (1998).

[5] Baldo, M. A. et al. Very high-efficiency green organic light-emitting devices based on electrophosphorescence. *Appl. Phys. Lett.* **75**, 4–6 (1999).

[6] Jeong, M. et al. Deep learning for development of organic optoelectronic devices: efficient prescreening of hosts and emitters in deep-blue fluorescent OLEDs. *npj Computational Mater.* **8**, 147 (2022).

[7] Gómez-Bombarelli, R. et al. Design of efficient molecular organic light-emitting diodes by a high-throughput virtual screening and experimental approach. *Nat. Mater.* **15**, 1120–1127 (2016). 27500805 10.1038/nmat4717

[8] Kwon, S. & Yoon, S. DeepCCI: end-to-end deep learning for chemical-chemical interaction prediction. Proceedings of the 8th ACM International Conference on Bioinformatics, Computational Biology, and Health Informatics. Boston, MA, USA: ACM, 2017, 203–212

[9] Joung, J. F. et al. Deep learning optical spectroscopy based on experimental database: potential applications to molecular design. *JACS Au* **1**, 427–438 (2021). 34467305 10.1021/jacsau.1c00035 PMC8395663

[10] Fang, X. M. et al. Geometry-enhanced molecular representation learning for property prediction. *Nat. Mach. Intell.* **4**, 127–134 (2022).

[11] Peng, S. P. & Zhao, Y. Convolutional neural networks for the design and analysis of non-fullerene acceptors. *J. Chem. Inf. Modeling* **59**, 4993–5001 (2019). 10.1021/acs.jcim.9b00732 31710212

[12] Barcza, S. et al. Structured biological data in the molecular access system. *J. Chem. Inf. Computer Sci.* **25**, 55–59 (1985).

[13] Rogers, D. & Hahn, M. Extended-connectivity fingerprints. *J. Chem. Inf. Modeling* **50**, 742–754 (2010). 10.1021/ci100050t 20426451

[14] Lecun, Y. et al. Gradient-based learning applied to document recognition. *Proc. IEEE* **86**, 2278–2324 (1998).

[15] Krizhevsky, A., Sutskever, I. & Hinton, G. E. ImageNet classification with deep convolutional neural networks. *Commun. ACM* **60**, 84–90 (2017).

[16] Lipton, Z. C., Berkowitz, J. & Elkan, C. A critical review of recurrent neural networks for sequence learning. Print at 10.48550/arXiv.1506.00019 (2015).

[17] Sperduti, A. & Starita, A. Supervised Neural Networks for the Classification of Structures. *IEEE Trans. Neural Netw. Learn. Syst.* **8**, 714 (1997). 10.1109/72.572108 18255672

[18] Wu, Z. H. et al. A comprehensive survey on graph neural networks. *IEEE Trans. Neural Netw. Learn. Syst.* **32**, 4–24 (2021). 32217482 10.1109/TNNLS.2020.2978386

[19] Bengio, Y., Courville, A. & Vincent, P. Representation learning: a review and new perspectives. Print at 10.48550/arXiv.1206.5538 (2012). 10.1109/TPAMI.2013.50 23787338

[20] Vaswani, A. et al. Attention is all you need. Print at 10.48550/arXiv.1706.03762 (2017).

[21] Devlin, J. et al. BERT: pre-training of deep bidirectional transformers for language understanding. Print at 10.48550/arXiv.1810.04805 (2018).

[22] Stewart, J. J. P. Optimization of parameters for semiempirical methods VI: more modifications to the NDDO approximations and re-optimization of parameters. *J. Mol. Modeling* **19**, 1–32 (2013). 10.1007/s00894-012-1667-x PMC3536963 23187683

[23] Stewart, J. J. P. Optimization of parameters for semiempirical methods I. Method. *J. Computational Chem.* **10**, 209–220 (1989).

[24] Hamilton, W. L., Ying, R. & Leskovec, J. Inductive representation learning on large graphs. Print at 10.48550/arXiv.1706.02216 (2017).

[25] Santoro, F. et al. Effective method for the computation of optical spectra of large molecules at finite temperature including the duschinsky and herzberg–teller effect: the Qx band of porphyrin as a case study. *J. Chem. Phys.* **128**, 224311 (2008). 18554017 10.1063/1.2929846

[26] Niu, Y. L. et al. Theory of excited state decays and optical spectra: application to polyatomic molecules. *J. Phys. Chem. A* **114**, 7817–7831 (2010). 20666533 10.1021/jp101568f

[27] Blum, L. C. & Reymond, J. L. 970 Million druglike small molecules for virtual screening in the chemical universe database GDB-13. *J. Am. Chem. Soc.* **131**, 8732–8733 (2009). 19505099 10.1021/ja902302h

[28] Ramakrishnan, R. et al. Electronic spectra from TDDFT and machine learning in chemical space. *J. Chem. Phys.* **143**, 084111 (2015). 26328822 10.1063/1.4928757

[29] Ruddigkeit, L. et al. Enumeration of 166 billion organic small molecules in the chemical universe database GDB-17. *J. Chem. Inf. Modeling* **52**, 2864–2875 (2012). 10.1021/ci300415d 23088335

[30] Hatakeyama, T. et al. Ultrapure blue thermally activated delayed fluorescence molecules: efficient HOMO-LUMO separation by the multiple resonance effect. *Adv. Mater.* **28**, 2777–2781 (2016). 26865384 10.1002/adma.201505491

[31] Zhang, Q. S. et al. Efficient blue organic light-emitting diodes employing thermally activated delayed fluorescence. *Nat. Photonics* **8**, 326–332 (2014).

[32] Xu, Y. C. et al. Constructing charge-transfer excited states based on frontier molecular orbital engineering: narrowband green electroluminescence with high color purity and efficiency. *Angew. Chem. Int. Ed.* **59**, 17442–17446 (2020). 10.1002/anie.202007210 32533603

[33] Xu, Y. C. et al. Highly efficient electroluminescent materials with high color purity based on strong acceptor attachment onto B–N-containing multiple resonance frameworks. *CCS Chem.* **4**, 2065–2079 (2022).

[34] Wang, Q. Y. et al. Precise functionalization of a multiple-resonance framework: constructing narrowband organic electroluminescent materials with external quantum efficiency over 40%. *Adv. Mater.* **35**, e2205166 (2023). 36325646 10.1002/adma.202205166

[35] Chen, T. Q. & Guestrin, C. XGBoost: a scalable tree boosting system. Proceedings of the 22nd ACM SIGKDD International Conference on Knowledge Discovery and Data Mining. San Francisco, CA, USA: ACM, 2016, 785–794.

[36] Xu, K. Y. L. et al. How powerful are graph neural networks? Print at 10.48550/arXiv.1810.00826 (2018).

[37] Xiong, Z. P. et al. Pushing the boundaries of molecular representation for drug discovery with the graph attention mechanism. *J. Medicinal Chem.* **63**, 8749–8760 (2020). 10.1021/acs.jmedchem.9b00959 31408336

[38] Schütt, K. T. et al. SchNet – A deep learning architecture for molecules and materials. *J. Chem. Phys.* **148**, 241722 (2018). 29960322 10.1063/1.5019779

[39] Maziarka, Ł. et al. Molecule attention transformer. Print at 10.48550/arXiv.2002.08264 (2020).

[40] Abadi, M. et al. TensorFlow: large-scale machine learning on heterogeneous distributed systems. Print at 10.48550/arXiv.1603.04467 (2016).

[41] Chen, J. F., Zhu, J. & Song, L. Stochastic training of graph convolutional networks with variance reduction. Print at 10.48550/arXiv.1710.10568 (2017).

[42] Krogh, A. & Hertz, J. A. A simple weight decay can improve generalization. Proceedings of the 4th International Conference on Neural Information Processing Systems. Denver, CO, USA: ACM, 1991, 950–957.

[43] Landrum, G. RDKit: Open-source cheminformatics software at https://www.rdkit.org (2016).

[44] Pracht, P., Bohle, F. & Grimme, S. Automated exploration of the low-energy chemical space with fast quantum chemical methods. *Phys. Chem. Chem. Phys.* **22**, 7169–7192 (2020). 32073075 10.1039/c9cp06869d

[45] Bannwarth, C. et al. Extended tight-binding quantum chemistry methods. *WIREs Computational Mol. Sci.* **11**, e1493 (2021).

[46] Bannwarth, C., Ehlert, S. & Grimme, S. GFN2-xTB—An accurate and broadly parametrized self-consistent tight-binding quantum chemical method with multipole electrostatics and density-dependent dispersion contributions. *J. Chem. Theory Comput.* **15**, 1652–1671 (2019). 30741547 10.1021/acs.jctc.8b01176

[47] Grimme, S., Bannwarth, C. & Shushkov, P. A robust and accurate tight-binding quantum chemical method for structures, vibrational frequencies, and noncovalent interactions of large molecular systems parametrized for all spd-block elements (*Z* = 1–86). *J. Chem. Theory Comput.* **13**, 1989–2009 (2017). 28418654 10.1021/acs.jctc.7b00118

[48] Frisch, M. J. et al. Gaussian 09 Revision D. 01. https://gaussian.com/g09citation/ (2016).

[49] Lu, T. & Chen, F. W. Quantitative analysis of molecular surface based on improved marching tetrahedra algorithm. *J. Mol. Graph. Model.* **38**, 314–323 (2012). 23085170 10.1016/j.jmgm.2012.07.004

[50] Lu, T. & Chen, F. W. Multiwfn: a multifunctional wavefunction analyzer. *J. Comput. Chem.* **33**, 580–592 (2012). 22162017 10.1002/jcc.22885

[51] Lu, T. & Chen, F. W. Atomic dipole moment corrected hirshfeld population method. *J. Theor. Comput. Chem.* **11**, 163–183 (2012).

[52] Lu, T. & Chen, F. W. Comparison of computational methods for atomic charges. *Acta Phys.-Chim. Sin.* **28**, 1–18 (2012).

[53] Lu, T. & Chen, Q. X. Independent gradient model based on Hirshfeld partition: a new method for visual study of interactions in chemical systems. *J. Comput. Chem.* **43**, 539–555 (2022). 35108407 10.1002/jcc.26812

