# Supplementary material for: Frontier molecular orbital weighted model based networks for revealing organic delayed fluorescence efficiency
Source: Light Sci Appl. 2025 Feb 10;14:75. doi: 10.1038/s41377-024-01713-w (PMC11808113; doi:10.1038/s41377-024-01713-w)
Supplement: Supplementary file 1 — Supplementary Information for Frontier Molecular Orbital Weighted Model Based Networks for Revealing Organic Delayed Fluorescence Efficiency [file 41377_2024_1713_MOESM1_ESM.docx]

**Supplementary Information**

**for**

**Frontier Molecular Orbital Weighted Model Based Networks for Revealing Organic Delayed Fluorescence Efficiency**

Zhaoming He,^1^ Hai Bi, ^1,*^ Baoyan Liang,^1^ Zhqiang Li,^1^ Heming Zhang,^1^ Yue Wang^1, 2, 3,*^

*^1^Jihua Laboratory, 28 Huandao South Road, Foshan, 528200, Guangdong Province, P. R. China.*

*^2^State Key Laboratory of Supramolecular Structure and Materials, College of Chemistry, Jilin University, 2699 Qianjin Avenue, Changchun, 130012, P. R. China*

*^3^Jihua Hengye Electronic Materials CO. LTD. Foshan 528200, Guangdong Province, P. R. China.*

*^*^ Authors to whom correspondence should be addressed:*

*bihai@jihualab.com (HB); yuewang@jlu.edu.cn* *(YW)*

**Contents**

**S1.** Synthetic route of DPQ-DPAC and DPQCN-DPAC

**S2.** Representation visualizations generated from reported methods

**S3.** Distribution of PLQY and variance of the attention weights

**S4.** Confirmation of supplementary molecular orbital contribution and vibration information

**S5.** Summarization of hyperparameters for ESIN

**S6.** Comparison of experimental results based on differential methods for PLQY predictions

**S7.** The information on TADF molecules with NAI-based acceptors

**S8.** The information on TADF molecules with quinoxalinyl acceptors

**S9.** PLQY values of DPQ-DPAC and DPQCN-DPAC

**S10**. Computational resources and process times

**References**

**S1. Synthetic route of DPQ-DPAC and DPQCN-DPAC**

**Synthesis of DPQ-DPAC:** 6-Bromo-2,3-diphenylquinoxaline (1.81 g, 5.00 mmol), 9,9-diphenyl-9,10-dihydroacridine (2.00 g, 6.00 mmol), cesium carbonate (2.93 g, 9.00 mmol), Pd_2_(dba)_3_ (137 mg, 0.15 mmol) and tri-tert-butylphosphine tetrafluoroborate (87 mg, 0.30 mmol) was dissolved in 25.0 mL toluene, which was heated to reflux under nitrogen for 8 h. When the reaction completed and cooled down to room temperature, the mixture was poured into dichloromethane (50.0 mL) and water (50.0 mL). The organic layer was collected after washing the mixture with dichloromethane for three times, the solvents were removed and the target product was purified by column chromatography. DPQ-DPAC was obtained as a bright green solid with a yield of 75.6% (2.32 g). ^1^H NMR (600 MHz, DMSO-*d*6) *δ* 8.38 (d, *J* = 8.7 Hz, 1H), 7.87 (d, *J* = 2.3 Hz, 1H), 7.55 – 7.33 (m, 17H), 7.32 – 7.27 (m, 2H), 7.12 (ddd, *J* = 8.4, 7.2, 1.5 Hz, 2H), 7.00 – 6.91 (m, 7H), 6.83 (dd, *J* = 7.8, 1.5 Hz, 2H), 6.52 (dd, *J* = 8.2, 1.1 Hz, 2H). ^13^C NMR (151 MHz, Chloroform-*d*) *δ* 153.60, 146.07, 142.44, 141.83, 141.73, 140.27, 138.45, 138.28, 132.78, 131.36, 130.92, 130.39, 130.15, 129.89, 129.25, 129.19, 128.41, 128.39, 127.72, 126.98, 126.44, 120.95, 114.91, 56.96. ESI-MS: m/z: 613.24 (calcd: 613.25).

**Synthesis of Intermediate 1:** 4-Ethynylbenzonitrile (2.54 g, 20.0 mmol) and 4-bromobenzonitrile (3.64 g, 20.0 mmol) was dissolved in 15.0 mL THF and 15.0 mL Et_3_N mixed solvents, Pd(PPh_3_)Cl_2_(0.42 g, 0.6 mmol) and CuI (0.38 g, 1.0 mmol) was added afterwards. The mixture was heated at 60 ℃ for 8 h. After the reaction was completed, the mixture was washed with dichloromethane (50.0 mL) and water (50.0 mL) for three times. The organic layer was collected and concentrated. The target product was purified by recrystallization with dichloromethane and methanol. Intermediate 1 was obtained as a white solid with a yield of 83.7% (3.82 g). ^1^H NMR (600 MHz, Chloroform-*d*) *δ* 7.68 (d, *J* = 8.2 Hz, 2H), 7.63 (d, *J* = 8.1 Hz, 2H). ESI-MS: m/z: 228.13 (calcd: 228.07).

**Synthesis of Intermediate 2:** Intermediate 2 was obtained by heating intermediate 1 (3.42 g, 15.0 mmol) in 50.0 mL DMSO at 150 ℃ under oxygen for 48 h. After the reaction was completed and cooled to room temperature, the mixture was poured into water. The precipitated solid was filtered under reduced pressure. The target product was purified by column chromatography. Yellow solid was obtained with a yield of 64.8% (2.53 g). ^1^H NMR (600 MHz, Chloroform-*d*) *δ* 8.15 – 8.08 (m, 2H), 7.88 – 7.82 (m, 2H). ESI-MS: m/z: 260.13 (calcd: 260.06).

**Synthesis of Intermediate 3:** Intermediate 2 (2.08 g, 8.0 mmol) and 4-bromobenzene-1,2-diamine (1.50 g, 8.0 mmol) was dissolved in 20.0 mL ethanol and 2.0 mL acetic acid, and the mixture was heated to reflux for 4 h. After the mixture cooled to room temperature, the mixture was filtrated to obtain white solid, which was washed with saturated sodium bicarbonate solution and cold ethanol. The target product was 3.08 g with a yield of 93.6%. ^1^H NMR (600 MHz, DMSO-*d*_6_) *δ* 8.49 (d, *J* = 2.2 Hz, 1H), 8.18 (d, *J* = 8.9 Hz, 1H), 8.11 (dd, *J* = 8.9, 2.2 Hz, 1H), 7.91 – 7.87 (m, 4H), 7.67 (dq, *J* = 8.3, 1.8 Hz, 4H). ESI-MS: m/z: 410.00 (calcd: 410.02).

**Synthesis of** **DPQCN-DPAC:** The synthesis of DPQCN-DPAC was similar with that of DPQ-DPAC by replacing 6-Bromo-2,3-diphenylquinoxaline with intermediate 3 (2.06 g, 5.0 mmol). The crude product was purified by column chromatography. DPQCN-DPAC was obtained as orange solid with a yield of 72.0% (2.39 g). ^1^H NMR (600 MHz, DMSO-*d*_6_) *δ* 8.43 (d, *J* = 8.8 Hz, 1H), 7.95 (d, *J* = 2.3 Hz, 1H), 7.93 – 7.87 (m, 4H), 7.73 – 7.65 (m, 4H), 7.52 (dd, *J* = 8.8, 2.3 Hz, 1H), 7.36 (dd, *J* = 8.2, 6.6 Hz, 4H), 7.33 – 7.27 (m, 2H), 7.12 (ddd, *J* = 8.4, 7.2, 1.5 Hz, 2H), 6.98 (ddd, *J* = 8.1, 7.3, 1.2 Hz, 2H), 6.95 – 6.92 (m, 4H), 6.84 (dd, *J* = 7.8, 1.5 Hz, 2H), 6.51 (dd, *J* = 8.2, 1.2 Hz, 2H). ^13^C NMR (151 MHz, Chloroform-*d*) *δ* 151.00, 150.92, 145.86, 143.77, 142.37, 142.21, 142.12, 141.53, 140.51, 133.80, 132.37, 131.57, 131.47, 130.53, 130.34, 130.26, 129.47, 127.73, 126.96, 126.49, 121.28, 118.15, 118.11, 115.06, 113.33, 113.25, 56.98. ESI-MS: m/z: 663.01 (calcd: 663.24).

**S2. Representation visualizations generated from reported methods**

**
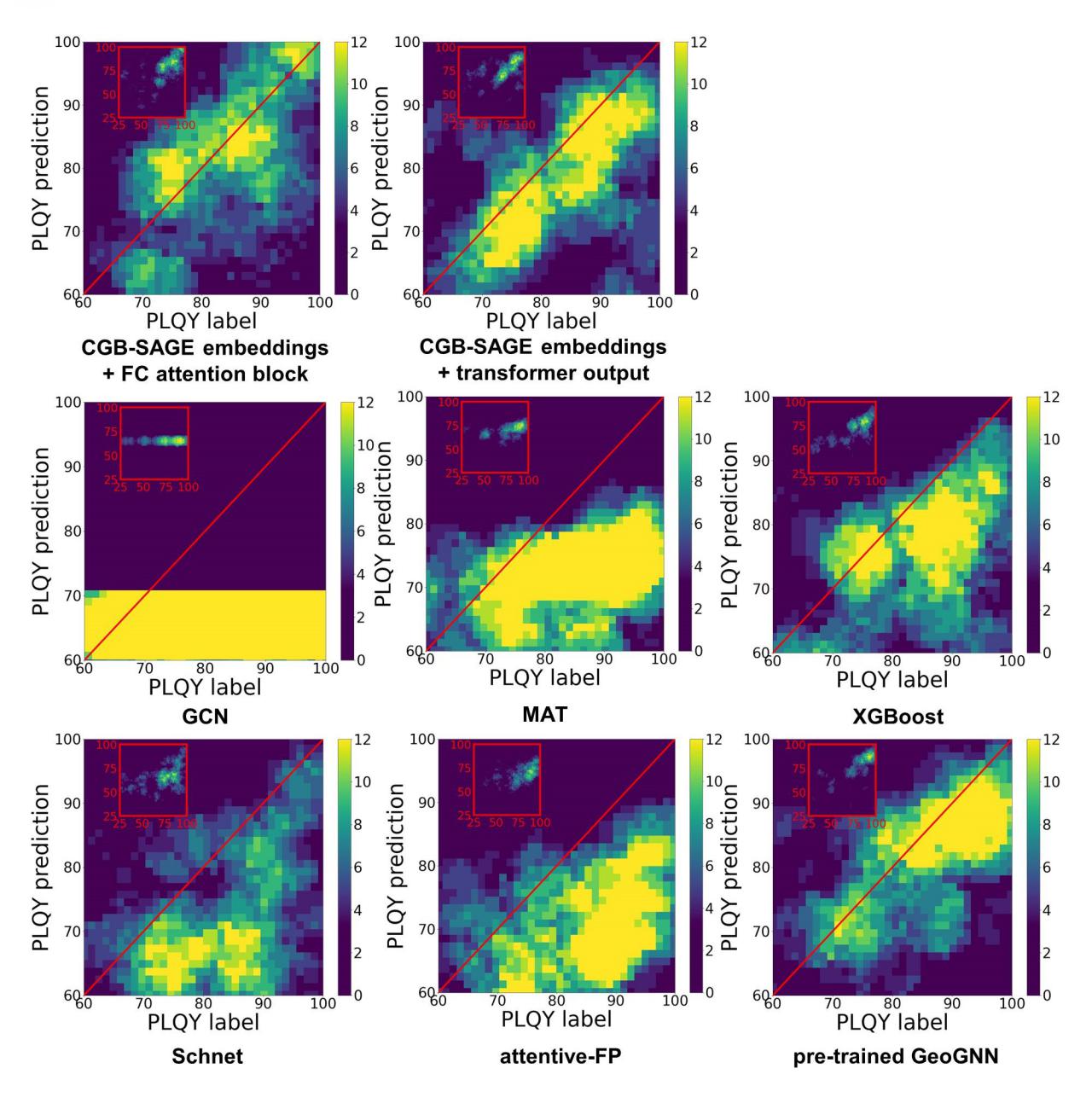
Fig. S1.** Representation visualizations generated from different methods that were reported for molecule properties prediction.

**Figure S1** presents the representation visualizations generated from recently reported state-of-the-art methods for molecule properties prediction based on the collected TADF data set with the same data partition configuration that was adopted in **ESIN**. We have verified the following 6 kinds of models: XGBoost^1^, GCN,^2^ attentive-FP,^3^ Schnet,^4^ MAT (Molecule Attention Transformer) ^5^ and pre-trained GeoGNN.^6^ For GCN and attentive-FP, adjacency matrix was used to obtain the embedding of molecules. As a variant of DTNNs, Schnet was incorporated with both atom representations and interatomic distances. The MAT adopts transformer block to build the atom-to-attribute relationship. In the experiments, the numbers of transformer block and sizes of the hidden layers were adjusted for the convergence. Specifically, the MAT model employs two transformers blocks of 2 heads with a hidden dimension of 256. The visualization parameters are identical to that displayed in **Figure 3b**. The results revealed that GCN model only predicts the mean value of the PLQY in data set, while XGBoost, attentive-FP, Schnet, MAT and pre-trained GeoGNN can only establish the relation between structure and the PLQY. The accuracies of the XGBoost, Schnet and attentive-FP are relatively lower than the pre-trained GeoGNN. For the reported models, on the lower right corner of the visualization the bright area is obviously larger, suggesting that there are more molecules with underestimated PLQYs.

**S3. Distribution of PLQY and variance of the attention weights**

**
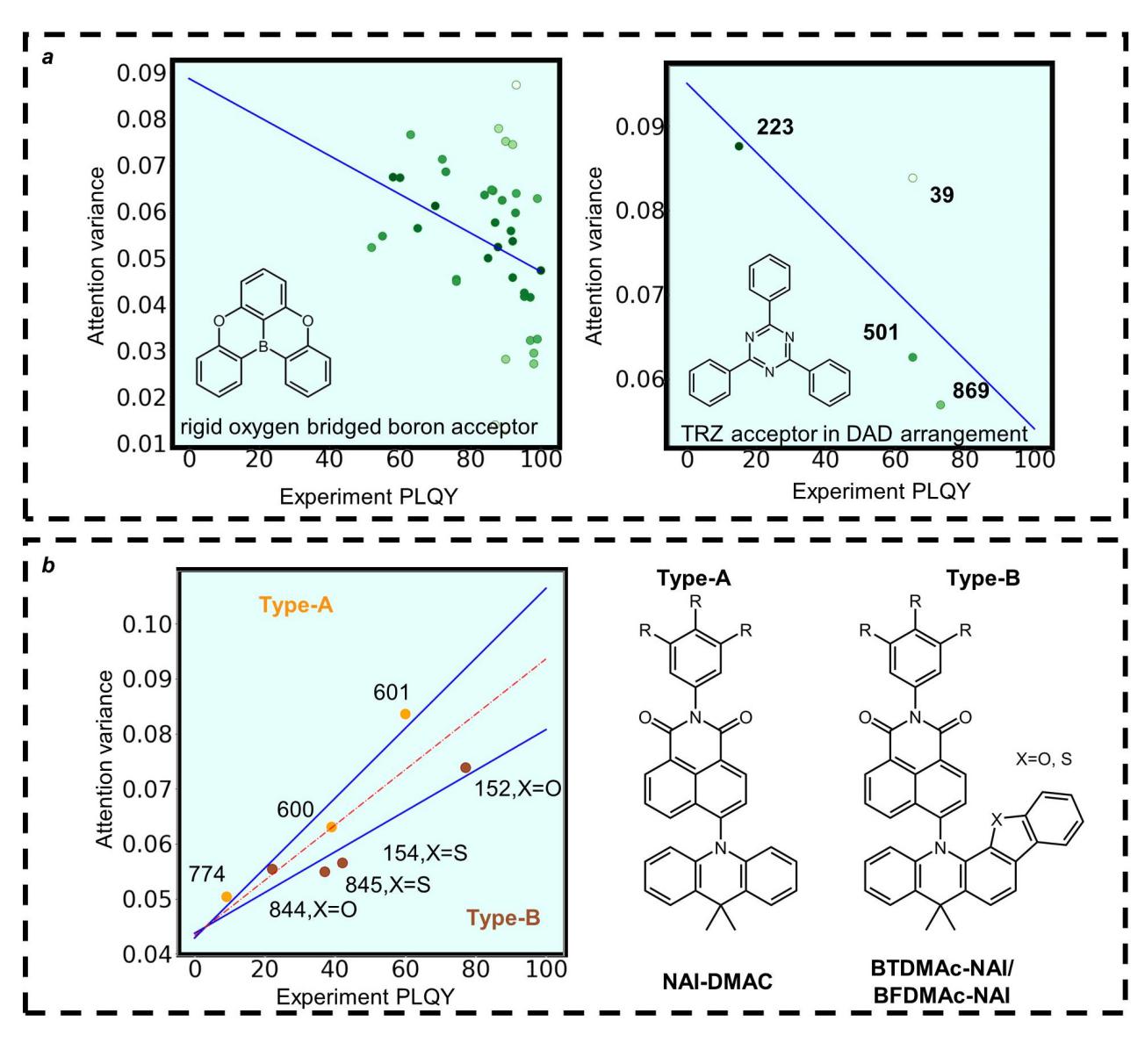
**

**Fig. S2.** (a) Distribution of PLQY and variance of the attention weights of molecules in the TADF data set with rigid oxygen bridged boron acceptor and TRZ acceptor-based TADF molecules with D-A-D arrangement. (b) Different distribution areas of TADF molecules with the same acceptor and two type donor units.

**Figure S2** presents the PLQY distribution and variance of the attention weights of the molecules with the similar acceptors in the TADF data set. A consistent relationship between the PLQYs and the variance of attention weights for the molecules based on similar acceptors and identical D-A arrangement.

**S4. Confirmation of supplementary molecular orbital contribution and vibration information**

**Table S1.** Performance improvement with additional molecular orbital contribution and vibration information.

| **Wavelength Prediction** | **ECFP with Extra Information** | **ECFP Only** |
| --- | --- | --- |
| Mean Error | **24.82** | 24.96 |
| Maximum Error | **229.27** | 234.19 |
| MAPE ^a^ (%) | **4.93** | 4.96 |

^a^ MAPE: minimum mean absolute percentage of error.

The data are randomly divided into 748 training examples and a testing set of 188 examples. The **Table S1** shows the experimental results of 100 repetitions based on a XGBoost model. The maximum predict error of experiments for 100 repetitions was defined as the maximum error. It was demonstrated that the molecule representation with simple supplementary molecular orbital contribution and vibration information can improve the performance of the prediction of the emission wavelength of the TADF emitters. The extra information includes the maximum contribution of four FMOs (HOMO-1, HOMO, LUMO and LUMO+1) and the maximum vibration frequency of the TADF molecule. The molecular orbital contribution information of the substructure centers and the vibration information are concatenated with the ECFPs of the TADF molecules, and the length of each ECFPs is 300 bits. Thresholds are created for these extra data to maintain consistency with the ECFPs. Specifically, the molecular orbital contribution over 30% is set to 1, the vibration frequency beyond 2000 cm^-1^ is set to 1, and others are set to 0.

**S5. Summarization of hyperparameters for ESIN**

**Table S2.** Summarization of hyperparameters for **ESIN**.

| Hyperparameter | FC attention blocks | Transformer output |
| --- | --- | --- |
| Training epoch | 45 | 65 |
| Weight decay | 0.0001 | 0.0001 |
| lr | 0.005 | 0.005 |
| Batch size | 32 | 32 |
| AdamW, β | (0.8,0.99) | (0.8,0.99) |
| CGB SAGE layers CGB SAGE neighbors | (4, (4,3,10,3)) | (4, (4,3,10,3)) |
| CGB SAGE hidden dimension | (64,128,128,128) | (64,128,128,128) |
| ECFPs bits, radius | (2048, 2) | (300, 2) |
| attention block hidden dimension | 128 | 812 |
| Dropout | 0 | 0 |

**S6. Comparison of experimental results based on differential methods for PLQY predictions**

**Table S3.** Comparison of experimental results based on differential methods for PLQY predictions.

| **Model** | **MAPE** | ***eff*_85_** | ***recall*_85_** |
| --- | --- | --- | --- |
| Attentive-FP | 0.91 | 0.4 | 0.04 |
| XGBoost + ECFP | 0.77 | 0.57 | 0.21 |
| Schnet | 0.78 | **0.68** | 0.21 |
| Pre-trained GeoGNN | 0.71 | 0.58 | 0.43 |
| ESIN FC Attention block | **0.63** | 0.61 | **0.47** |
| ESIN transformer output | **0.63** | 0.63 | 0.33 |

The main purpose of the PLQY predictions of is to figure out the potential candidate molecules that may have high PLQYs. During conducting a virtual screening, a target criterion would be to require that PLQY$\geq\theta$%*.* Here, we proposed the screening efficiency and the recall rate as two performance indexes for the evaluation of the **ESIN**.

$$eff_{\theta}=\frac{TP_{\geq\theta}}{TP_{\geq\theta}+FP_{\geq\theta}}$$

$$recall_{\theta}=\frac{TP_{\geq\theta}}{TP_{\geq\theta}+FN_{\geq\theta}}$$

In the above formula, the $eff_{\theta}$ represents the screening efficiency, where the subscript *$\theta$* refers to the screening criterion. The term *TP*_≥θ_ means the true positives of the prediction results, and the *FP*_≥θ_ is the false positives. The $recall_{\theta}$ is defined as the percentage of correctly predicted molecules from all molecules satisfied the screening criterion in the test set.

It is worth to note that the data partitions for training XGBoost, GCN, attentive-FP, and Schnet are identical to that for **ESIN**, while 3 molecules in the data set are removed for training pre-trained GeoGNN and MAT due to the pre-processing requirements. The details of prediction results are shown in **Table S3**. The best performance results are highlighted with bold style. Our DL model **ESIN** achieved the minimum mean absolute percentage of error (MAPE) and the best *recall_85_*. The **ESIN** with FC attention block is second only to Schnet on *eff_85_*. The results of the comprehensive experiments demonstrated that the pre-trained GeoGNN can also achieve high recall, which may be due to that the pre-trained GeoGNN has a refined chemical bond feature extraction characteristic and the pre-training process may be beneficial to improving accuracy of prediction. In addition, the poor performance of attentive-FP can be attributed to the loss of structural information when only using adjacency matrix to represent large molecules.

**S7. The information on TADF molecules with NAI-based acceptors**

**Table S4.** Molecules with NAI as a rigid electron acceptor moiety.

|  | **SMILES** | **PLQY** | **ID** |
| --- | --- | --- | --- |
| 1 | CC(C)C1=CC=C(C2=CC=C(N3C(=O)C4=CC=CC5=C(N6C7=C(C=CC=C7)C(C)(C)C7=C6C6=C(C=C7)C7=C(C=CC=C7)O6)C=CC(=C45)C3=O)C=C2)C=C1 | 77.0 | 152 |
| 2 | CC(C)C1=CC=C(C2=CC=C(N3C(=O)C4=CC=CC5=C(N6C7=C(C=CC=C7)C(C)(C)C7=C6C6=C(C=C7)C7=C(C=CC=C7)S6)C=CC(=C45)C3=O)C=C2)C=C1 | 42.0 | 154 |
| 3 | CC1(C)C2=CC=CC=C2N(C2=CC=C3C(=O)N(C4=CC=CC=C4)C(=O)C4=C3C2=CC=C4)C2=CC=CC=C21 | 38.9 | 600 |
| 4 | CC(C)(C)C1=CC=C(N2C(=O)C3=CC=CC4=C(N5C6=CC=CC=C6C(C)(C)C6=CC=CC=C65)C=CC(=C34)C2=O)C=C1 | 59.9 | 601 |
| 5 | CC(C)(C)C1=CC(N2C(=O)C3=CC=CC4=C(N5C6=CC=CC=C6C(C)(C)C6=CC=CC=C65)C=CC(=C34)C2=O)=CC(C(C)(C)C)=C1 | 9.0 | 774 |
| 6 | O=C1C2C=CC(N3C4=C(C(C)(C5=C3C3SC6=C(C=CC=C6)C=3C=C5)C)C=CC=C4)=C3C=2C(C(N1C1C=CC(C(C)(C)C)=CC=1)=O)=CC=C3 | 22.0 | 844 |
| 7 | O1C2=C(C=CC=C2)C2C=CC3=C(N(C4=C5C6=C(C(N(C7C=CC(C(C)(C)C)=CC=7)C(C6=CC=C5)=O)=O)C=C4)C4=C(C3(C)C)C=CC=C4)C=21 | 37.0 | 845 |

**S8. The information on TADF molecules with 2,3-diphenylquinoxaline acceptors**

**Table S5.** Molecules with 2,3-diphenylquinoxaline acceptor in the data set.

|  | **SMILES** | **PLQY** | **ID** | **Measurement** | **Ref.** |
| --- | --- | --- | --- | --- | --- |
| 1 | C1=CC2=C(C=C1)N(C1=CC=C(C3=NC4=CC5=C(C=C4N=C3C3=CC=C(N4C6=C(C=CC=C6)OC6=C4C=CC=C6)C=C3)OC3=C(C4=C(C=C3)CCCC4)C3=C4CCCCC4=CC=C3O5)C=C1)C1=C(C=CC=C1)O2 | 92 | 122 | Doped CBP films | [7] |
| 2 | CC1(C)C2=C(C=CC=C2)N(C2=C(F)C=C3N=C(C4=CC=C(C#N)C=C4)C(C4=CC=C(C#N)C=C4)=NC3=C2)C2=C1C=CC=C2 | 87 | 448 | 1% doped  Polystyrene films | [8] |
| 3 | CC1(C)C2=CC=CC=C2N(C2=CC=C3N=C(C4=CC=C(C#N)C=C4)C(C4=CC=C(C#N)C=C4)=NC3=C2)C2=CC=CC=C21 | 34 | 720 | **Crystal** | **[9]** |
| 4 | COC1=CC=C2C(=C1)CCC1=CC(OC)=CC=C1N2C1=CC=C(C2=NC3=CC=CC=C3N=C2C2=CC=C(N3C4=CC=C(OC)C=C4CCC4=CC(OC)=CC=C43)C=C2)C=C1 | 31 | 726 | Degassed CH2Cl2 solution | [10] |
| 5 | N(C1C=CC=CC=1)(C1C=CC=CC=1)C1=CC=C(C=C1)C1C(=NC2=C(N=1)C(C#N)=CC=C2C#N)C1C=CC(=CC=1)N(C1C=CC=CC=1)C1=CC=CC=C1 | 80 | 809 | Doped CBP films | [11] |
| 6 | N1=C2C(=NC(=C1C1=CC=C(C=C1)N1C3=C(C=C(C=C3)C(C)(C)C)C3C=C(C(C)(C)C)C=CC1=3)C1=CC=C(C=C1)N1C3=C(C=C(C=C3)C(C)(C)C)C3C=C(C(C)(C)C)C=CC=31)C=C(C#N)C(C#N)=C2 | 93 | 852 | Doped polystyrene films | [12] |
| 7 | N1C(=C(N=C2C=1C=C(C#N)C(C#N)=C2)C1=CC=C(C=C1)N1C2C=CC=CC=2C(C)(C2C=CC=CC=21)C)C1=CC=C(C=C1)N1C2=C(C=CC=C2)C(C2C=CC=CC=21)(C)C | 91 | 853 | Doped polystyrene films | [12] |
| 8 | N1=C2C(=NC(=C1C1=CC=C(C=C1)N1C3=C(C=C(C=C3)C(C)(C)C)C3C=C(C(C)(C)C)C=CC1=3)C1=CC=C(C=C1)N1C3=C(C=C(C=C3)C(C)(C)C)C3C=C(C(C)(C)C)C=CC1=3)C(C#N)=CC=C2C#N | 96 | 854 | Doped polystyrene films | [12] |
| 9 | N1=C2C(=NC(=C1C1=CC=C(C=C1)N1C3=C(C=CC=C3)C(C)(C3C=CC=CC1=3)C)C1=CC=C(C=C1)N1C3=C(C=CC=C3)C(C3C=CC=CC=31)(C)C)C(C#N)=CC=C2C#N | 72 | 855 | Doped polystyrene films | [12] |
| 10 | N1=C(C2=CC=C(C=C2)N2C3C=CC=CC=3OC3C2=CC=CC=3)C(C2C=CC(N3C4C(OC5=C3C=CC=C5)=CC=CC=4)=CC=2)=NC2C1=CC=C(C=2)C(=O)C1C=CC=CC=1 | 88 | 1031 | Doped film | [13] |
| 11 | N1C2C(=CC(C#N)=C(C=2)C#N)N=C(C2C=CC(=CC=2)N2C3=C(C=CC=C3)C3C4=C(C5=C(C=CC=C5)N4C4C=CC=CC=4)C4N(C5C=CC=CC=5C=4C=32)C2=CC=CC=C2)C=1C1=CC=CC=C1 | 73 | 1034 | Doped polystyrene films | [14] |
| 12 | N1C2C(N=C(C=1C1C=CC=CC=1)C1C=CC(N3C4=C(C=CC=C4)C4C5=C(C6=C(C=CC=C6)N5C5C=CC=CC=5)C5=C(C6C=CC=CC=6N5C5C=CC=CC=5)C=43)=CC=1)=C(C#N)C=CC=2C#N | 40 | 1035 | Doped polystyrene films | [14] |
| 13 | N1=C2C(=NC(=C1C1=CC=CC=C1)C1=CC=C(C=C1)C1(CC=CC=C1)C1=CC=C(C=C1)C1=C(C3C=CC=CC=3)N=C3C(=N1)C=C(C#N)C(C#N)=C3)C=C(C(C)=C2)C | 70 | 1068 | Doped film | [15] |

Note: Because the structure and D-A arrangement features of the molecule ID-122 and the molecule ID-1068 are obviously different from that of other molecules, the two molecules were not been adopted in the experiment.

**S9. PLQY values of DPQ-DPAC and DPQCN-DPAC**

**Table S6.** Experimental PLQY values of DPQ-DPAC and DPQCN-DPAC in solution (toluene: 1.0×10^-5^ M) and doped films with different doped concentrations (host: CBP).

|  | Solution(N_2_) | 5% | 10% | 20% | 100% |
| --- | --- | --- | --- | --- | --- |
| DPQ-DPAC | 0.137 | 0.839 | 0.800 | 0.833 | 0.320 |
| DPQCN-DPAC | 0.506 | 0.974 | 0.979 | 0.875 | 0.737 |

**S10. Computational resources and process times**

| Model | Training (min/epoch) | Testing (s/item) |
| --- | --- | --- |
| ESIN with FC attention blocks | 16.4 | 0.58 |
| ESIN with Transformer output layer | 16.6 | 0.57 |
|  | | |
| **Resources** | **Specification** | |
| CPU | Intel(R) Xeon(R) Platinum 8163 CPU @ 2.50GHz | |
| GPU | NVIDIA GeForce RTX 3090 Ti | |
| RAM | 128GB | |
| OS | Ubuntu 20.04.4 LTS | |

**References**

1. Chen, T., Guestrin, C. XGBoost: A Scalable Tree Boosting System. In: *Proceedings of the 22nd ACM SIGKDD International Conference on Knowledge Discovery and Data Mining*) (2016).

2. Xu, K., Hu, W., Leskovec, J., Jegelka, S. J. a. e.-p. How Powerful are Graph Neural Networks? Preprint at <https://doi.org/10.48550/arXiv.1810.00826> (2018).

3. Xiong, Z. et al. Pushing the Boundaries of Molecular Representation for Drug Discovery with the Graph Attention Mechanism. *J. Med. Chem.* **63**, 8749-8760 (2019).

4. Schütt, K. T., Sauceda, H. E., Kindermans, P. J., Tkatchenko, A., Müller, K. R. SchNet – A deep learning architecture for molecules and materials. *J. Chem. Phys.* **148**, 241722 (2018).

5. Maziarka, Ł., Danel, T., Mucha, S., Rataj, K., Tabor, J., Jastrzębski, S. J. a. e.-p. Molecule Attention Transformer. Preprint at <https://doi.org/10.48550/arXiv.2002.08264> (2020).

6. Fang, X. et al. Geometry-enhanced molecular representation learning for property prediction. *Nat. Mach. Intell.* **4**, 127-134 (2022).

7. Xie, F. M., Zhou, J. X., Zeng, X. Y. et al. Efficient circularly polarized electroluminescence from chiral thermally activated delayed fluorescence emitters featuring symmetrical and rigid coplanar acceptors. *Adv. Opt. Mater.* **9(9)**: 2100017(2021).

8. Kothavale, S., Chung, W. J., & Lee, J. Y. Rational molecular design of highly efficient yellow-red thermally activated delayed fluorescent emitters: a combined effect of auxiliary fluorine and rigidified acceptor unit. *ACS Appl. Mater. Interfaces*, **12(16)**, 18730-18738(2020).

9. Zheng, K., Ni, F., Chen, Z., Zhong, C., & Yang, C. Polymorph‐Dependent Thermally Activated Delayed Fluorescence Emitters: Understanding TADF from a Perspective of Aggregation State. *Angew. Chem. Int. Ed.* **59(25)**, 9972-9976 (2020).

10. Pashazadeh, R., Pander, P., Bucinskas, A., Skabara, P. J., Dias, F. B., & Grazulevicius, J. V. An iminodibenzyl–quinoxaline–iminodibenzyl scaffold as a mechanochromic and dual emitter: donor and bridge effects on optical properties. *Chem. Commun.* 54(98), 13857-13860 (2018).

11. Li, Z., Yang, D., Han, C., Zhao, B. et al. Optimizing Charge Transfer and Out‐Coupling of A Quasi‐Planar Deep‐Red TADF Emitter: towards Rec. 2020 Gamut and External Quantum Efficiency beyond 30%. *Angew. Chem. Int. Ed.* **60(27)**, 14846-14851 (2021).

12. Kothavale, S., Lee, K. H., & Lee, J. Y. Isomeric quinoxalinedicarbonitrile as color-managing acceptors of thermally activated delayed fluorescent emitters. *ACS Appl. Mater. Interfaces*, **11(19)**, 17583-17591 (2019).

13. Liang, J., Li, C., Cui, Y., Li, Z., Wang, J., Wang, Y. Rational design of efficient orange-red to red thermally activated delayed fluorescence emitters for OLEDs with external quantum efficiency of up to 26.0% and reduced efficiency roll-off. *J. Mater. Chem. C*‌‌ **8(5)**, 1614-1622 (2020).

14. Kothavale, S., Chung, W. J., Lee, J. Y. High efficiency and long lifetime orange-red thermally activated delayed fluorescent organic light emitting diodes by donor and acceptor engineering. *J. Mater. Chem. C* **9(2)**, 528-536 (2021).

15. Li, H. et al. Highly Efficient Orange‐Red Thermally Activated Delayed Fluorescence Compounds Comprising Dual Dicyano‐Substituted Pyrazine/Quinoxaline Acceptors. *ChemPlusChem*, **86(1)**, 95-102 (2021).
